# Alternative models of funding curiosity-driven research

**DOI:** 10.1073/pnas.2401237121

**Published:** 2025-01-27

**Authors:** Gerd Gigerenzer, Colin Allen, Stefan Gaillard, Robert L. Goldstone, Julia Haaf, William R. Holmes, Yoshihisa Kashima, Benjamin Motz, Sebastian Musslick, Angelika Stefan

**Affiliations:** ^a^Max Planck Institute for Human Development, Berlin 14195, Germany; ^b^Department of Philosophy, University of California, Santa Barbara, CA 93106; ^c^Institute for Science in Society, Radboud University, Nijmegen 6500 HC, Netherlands; ^d^Department of Psychological and Brain Sciences, Indiana University, Bloomington, IN 47405; ^e^Cognitive Science Program, Indiana University, Bloomington, IN 47405; ^f^Department of Psychological Methods, Statistics and Evaluation, University of Potsdam, Potsdam 14476, Germany; ^g^Melbourne School of Psychological Sciences, The University of Melbourne, Melbourne, VIC 3010, Australia; ^h^Department of Cognitive, Linguistic and Psychological Sciences, Brown University, Providence, RI 02912; ^i^Department of Psychology, University of Liverpool, Liverpool L69 7ZA, United Kingdom

**Keywords:** metascience, science funding, curiosity-driven research

## Abstract

Funding of curiosity-driven science is the lifeblood of scientific and technological innovation. Various models of funding allocation became institutionalized in the 20th century, shaping the present landscape of research funding. There are numerous reasons for scientists to be dissatisfied with current funding schemes, including the imbalance between funding for curiosity-driven and mission-directed research, regional and country disparities, path-dependency of who gets funded, gender and race disparities, low inter-reviewer reliability, and the trade-off between the effort and time spent on writing or reviewing proposals and doing research. We discuss possible alternative models for dealing with these issues. These alternatives include incremental changes such as placing more weight on the proposals or on the investigators and representative composition of panel members, along with deeper reforms such as distributed or concentrated funding and partial lotteries in response to low inter-reviewer reliability. We also consider radical alternatives to current funding schemes: the removal of political governance and the introduction of international competitive applications to a World Research Council alongside national funding sources. There is likely no single best way to fund curiosity-driven research; we examine arguments for and against the possibility of systematically evaluating alternative models empirically.

Funding of curiosity-driven research is the lifeblood of scientific innovation, but how and by whom research is funded has varied a great deal in history. Galileo Galilei was funded by the Medici family; Isaac Newton relied on his resources as a professor in Cambridge; Johannes Kepler constantly complained that he had no money; Charles Darwin spent his father’s inheritance and enlisted his family as research assistants; Marie Curie benefitted from the subsidies of wealthy French benefactors. Organized public funding is a latecomer: The NIH in the United States was founded in 1949, the NSF in 1950, and the European Research Council (ERC) in 2007. To improve current funding models, a systematic review of the evolved practices and experimentation with new ideas is indispensable. For instance, the US “CHIPS and Science Act of 2022” charged the NSF with considering alternatives to and experimenting within NSF’s processes for soliciting, reviewing, and funding research.

In this article, we focus primarily on funding in the North American, European, and Australasian contexts and ask: What issues are inherent to current models for funding curiosity-driven research? How might established funding schemes be improved? What radical alternative models of funding should be considered? Our goal is not to exhaustively identify issues or argue for a single best funding policy. Rather, we highlight the spectrum of issues and alternatives and consider the possibility of studying their relative advantages empirically.

## Funding for Curiosity-Driven Science and Its Issues

In this article, we deal with curiosity-driven science, that is, research that is driven by the curiosity of scientists who select for themselves which research questions and methods of investigation they wish to pursue and to propose for funding by state or private research foundations. We distinguish curiosity-driven research from mission-directed research (1), in which government, industry, or nonprofit agencies engage researchers or incentivize multiple research groups to work on an agency-specified topic, possibly also with agency-specified methods. This distinction, based on who selects the research questions, is less vague than other distinctions such as between basic and applied research, or those based on the time horizon for the first expected applications. It is often impossible to predict whether and when scientific research will result in important applications. For instance, curiosity-driven research on mRNA vaccines for cancer was funded by the ERC years before the COVID-19 pandemic but was quickly adapted to the unforeseen situation once it arose. Curiosity-driven research embodies the fertility of scientific imagination when carrying out research, the importance of serendipity in discovery, and the independence of science.

In what follows, we do not deal with the day-to-day issues of funding agencies, such as raising funds and finding reviewers, although the relative ease or difficulty of either clearly influences the kinds of research that get funded. Instead, we focus on the funding allocation models behind the daily work. We identify six issues facing current funding schemes and then discuss possible funding reforms and alternatives.

## Issues

### Discrepancy Between Funding Curiosity-Driven Science and Mission-Directed Research.

Most research funding goes to mission-directed research. For instance, Horizon Europe, the EU’s current key funding program for research and innovation, has a budget of 95.5 billion euros. Only 17% of Horizon Europe funding is allocated to curiosity-driven research, constituting the entire budget of the ERC; the other 83% supports mission-directed research whose goals are specified by governments and industry. This imbalance in allocation means that more than a third of all top-rated curiosity-driven research proposals cannot be funded by the ERC (2). Nevertheless, 40% of ERC projects were subsequently cited in patent applications (3), establishing the practical importance of curiosity-driven research. In the United States, there similarly exists a nearly six-to-one ratio in funding offered by the NIH and NSF, largely because of the significant amount allocated by NIH to mission-directed research. Increasing emphasis at NSF on “translational” research, represented by its Directorate for Technology and Innovative Partnerships (TIP) created in 2022 by the CHIPS and Science Act, indicates to many observers that it too is shifting toward more mission-driven funding opportunities. Australian funding for curiosity-driven research has declined as a proportion of GDP over the past several years, while mission-directed funding has remained politically popular and better funded (4).

Although corporations likewise tend to focus on mission-directed research, some have recognized that curiosity-driven research is important for innovation. In 1948, the Minnesota Mining and Manufacturing Company (3M) was one of the first corporations to explicitly allow scientists and engineers to spend 15% of their work time trying to invent anything they like. Google also provided one day a week for researcher-driven innovation, leading, among other things, to the invention of Gmail and Google Earth (5).

Investing more in curiosity-driven research might well lead to more innovation, although it is debatable what should be considered as a good measure of success. Does the quantity of publications and patents provide appropriate measures of funding success, especially given that only few are widely cited and used (6)? Some argue that the patent system impedes innovation by protecting monopolies and promoting litigation. Others argue that patents are precisely the drivers of innovation and the spread of the products of science (7). Yet government funding of curiosity-driven science is under threat from politicians, not only in the US Congress, where many argue that too much taxpayer money is spent on curiosity-driven science and that governments should leave it to companies to finance mission-directed research, but also in the United Kingdom and other countries (8). It is crucial to note that, while funding of mission-driven research has often achieved its goal, public funding of curiosity-driven research was instrumental in delivering innovations that could not have been imagined at the time the research was conducted. The relatively long and unpredictable timelines for the benefits of curiosity-driven research to become manifest contribute to the difficulty of designing measures of success that are suited to shorter-term politically or commercially driven funding cycles. Historical case studies may help establish the general value of curiosity-driven research as generating public value (9) although this approach cannot provide a direct measure of the value of recent and current research.

### Path Dependence: The Matthew Effect.

The Matthew effect refers, in the present context, to the phenomenon that a researcher who has previously been awarded grants is more likely to get another one. This path dependence can lead to a feedback loop that overamplifies actual differences between researchers. Equally qualified proposals by less well-known researchers are overlooked. Awarding funds to scientists who already have obtained considerable resources can also foster hypercompetitiveness and rivalry among the most successful grant applicants, supported by university administration that has become dependent on the indirect costs from grants (sometimes also incentivizing bad scientific practices), and can demotivate scientists who initially experience lower success (10, 11).

One study documenting this path dependence in detail showed that winners of a funding competition who were just above the funding threshold subsequently accumulated more than twice as much funding than did applicants just below that threshold. This path dependence of funding is driven both by winners having more resources to devote to applying for additional funding and by losers ceasing to compete (11).

One may reasonably argue that some degree of the Matthew effect should be expected given actual quality differences among researchers and that rewarding the best researchers is appropriate and therefore beneficial to science. However, the degree to which this effect exists possibly surpasses what is warranted by such quality differences. The Matthew effect can reinforce the discrepancies discussed in the next two sections.

### Country Disparities: Researchers in Many Countries Have Little Access to Funding.

The existence of regional disparities in funding received is by now well established. It is particularly salient with respect to differences between the Global South and Europe/North America/Australia. Yet the even larger disparities among individual countries within these regions or within subnational regions are rarely discussed.

Consider Europe, for which data are available regarding the vast inequalities that exist. Researchers in countries such as Bulgaria, Greece, Hungary, Portugal, Poland, and Romania receive relatively little support for curiosity-driven research from their own countries, and their chance of obtaining international grants is surprisingly low. To illustrate, in 2020, 21 young researchers from Belgium and 42 from The Netherlands were awarded ERC starting grants, compared with only one from Romania and three from Poland (even though the latter countries have larger populations). In fact, about half the member states of the EU receive extremely little from the funding pie. The northwestern countries in Europe, led by the United Kingdom, Germany, and France, collect most of the funds, with little left for potential talents in eastern or southeastern European countries. The disparity is evident in both the lower numbers of applications and the lower success rates. Nevertheless, the picture is not as straightforward as this may suggest. According to the ERC data, Cyprus and Slovenia have both received more funding per capita than any other EU countries, in part due to specific programs put in place by their national bodies to foster successful applications. Marked disparities exist not only between countries but also within the same country. For example, the UK governmental research council has spent four times more per capita in England than in Wales (12). Some researchers from underfunded regions or countries did receive funding as expatriates, but this further exacerbates discrepancies by contributing to a brain drain from the underfunded countries.

Although an initial estimate of the amount of country and regional disparities is provided by the lower number of applications and the lower success rate of the applications, these measures do not capture the full extent of geographic disparities, nor its causes such as the lack of local infrastructure and research capacities. The extent to which talented researchers from a large number of countries or regions are not given the opportunity to develop their ideas thus remains understudied.

In the United States, the NSF’s Established Program to Stimulate Competitive Research (EPSCoR) is designed to address disparities in science funding between different states. In 2022, over four decades after its origin in 1979, 25 US states remained eligible for EPSCoR funding, and it was still the subject of extensive debate both about whether the program had achieved its goals and about whether doing so would actually prove more harmful than beneficial to national scientific capacity (13).

### Gender and Racial Disparities.

Despite affirmative action and other attempts to promote gender equality, women continue to receive a smaller share of funding than men and have been underrepresented in reviewer panels (14). In the past decade, funding agencies have changed their procedures to address this issue, but results are mixed. Two phenomena are reported in most studies looking at gender disparities. First, women in academia apply for fewer grants than their male counterparts, even when controlling for the smaller number of female researchers in some fields, and they also request smaller amounts of money. Second, their success rate is similar to that for men, unless the focus of the grant review is on the quality of researchers as opposed to the quality of the proposal (15–17). For instance, an analysis of 6,319 grant applications to the NIH and multiple other agencies by the faculty at eight institutions affiliated with Harvard Medical School showed that only 26% of grant requests were submitted by women, but after controlling for academic rank, their success rate was the same as for men (18). Similarly, between 2015 and 2020, 34 to 38% of applications for ERC starting grants came from women, with no differences between success rates of male and female applicants. In 11 European countries plus Israel and Canada, the median percentage of women among all grant applicants was 42.5%, and again, no difference in success rate could be found (19). Reviews of the German Research Foundation (DFG) show similar results (20). However, one cannot infer equality of treatment from these comparable success rates because women surviving the gauntlet of barriers to reach that rank could plausibly have a higher quality than the average male at that rank. Also, studies from Canada (15), the Netherlands (16), and Belgium (21) indicate anti-female bias in evaluations of applications where the focus is on the principal investigator, as is often the case with late-career research funding programs. Moreover, average data mask systematic differences in gender participation between research fields, particularly in physics and engineering, such that field-specific gender disparity often remains undetected.

Disparities in funding in the United States have also been reported for Black principal investigators. After a study showed that the success rate of Black applicants to get an NIH R01 grant in 2000–2006 was only 16.1%, compared to 29.3% for White applicants, the NIH studied potential reasons and introduced its Enhancing Peer Review process. Yet one decade later, in 2014–2016, the gap persisted, with a success rate of 10.2% and 18.5%, respectively (22).

The root of gender and racial disparities in research funding cannot be attributed simply to a difference in application success, but likely reflects a combination of preexisting barriers to entering and remaining in academia, barriers to career progression, and negative stereotypes. For gender, such barriers include different societal expectations for men and women with respect to childcare, and insufficient support (at least in the United States) for parental leave (23).

### Low Reviewer Reliability.

Studies of reviewers’ evaluation of proposals indicate that the ability of review panels to rank applications is best within the very top and lower-to-middle range but is weak in the critical range of acceptance. For instance, an analysis of 130,000 NIH grants reported that a peer review score that is one standard deviation below the mean score of awarded grants is associated with 15% fewer citations, 19% fewer high-impact publications, and 14% fewer follow-up patents (24). Yet a reanalysis of the study reported that this predictability is limited to the top 2% of scores, whereas there is essentially no signal in the 2% to 20% range (AUC = 0.54 for grants with scores in the 20th percentile or better) (25). One possible inference from these two studies is that the peer review process is able to identify the top 2%, as well as those who underperform, but is not predictive in the 2% to 20% band. An alternative explanation for agreement on the 2% is the Matthew effect, which we discussed before. Nevertheless, the 2% to 20% region is critical because it spans the typical cutoff of the NIH and other funding agencies, which is between the top 10% and 20%. If agencies funded more than the top 20%, this lack of discriminability would be less consequential.

One possible reason for this limited predictability is low agreement among reviewers in evaluating the same proposal (26–29). Discussion among panel members is associated with increased agreement within a panel but reduced agreement between panels (30). Low predictability in the critical funding region opens the door to other factors that determine who is awarded a grant, including the Matthew effect. The unreliability of peer review introduces uncertainty to the selection process, prompting some researchers to describe the current system as “a lottery without the benefits of randomness” (31).

A possible counterargument to the claim about reviewer unreliability is that short-term measures of research success that lead to such judgments are themselves flawed. However, as briefly discussed in Discrepancy between funding curiosity-driven science and mission-directed research above, there are no well-attested methods for evaluating the benefits of curiosity-driven research in the long term, and it is possible, perhaps even likely, that even when assessed against long-term measures or research success, reviewer reliability would remain very low. The companion piece in this issue by Aczel et al. provides a detailed discussion of the present and future of peer review (32).

### The Trade-Off Between Writing Proposals and Managing Grants Versus Doing Research.

The grant proposal system requires researchers to devote substantial time to writing proposals that could have been used to conduct research. A survey reported that principal investigators spend on average 116 hours per NSF proposal (33). As proposal success rates decrease, the loss of research time increases. After one grant is awarded, researchers often have to begin applying for new grants rather than fully concentrate on the research to be done. In addition, professional pressures can motivate researchers to seek funding for reasons that extend beyond the value of the research itself, such as promotion and prestige. These external incentives are promoted by administrators who rely on grant success as the primary measure of achievement, in part because indirect costs have become a key source of funding for administrative initiatives. The large overhead costs of grants also increase the administrative pressures on researchers (and arguably overburden taxpayers) and divert the efforts of successful researchers toward lab management and other administrative tasks, constituting a form of brain drain.

### Potential Reforms.

There are many other potential issues that we do not have the space to address here. In what follows, we outline alternatives to present funding systems that might solve one or more of the six issues described above. We distinguish between incremental reforms that can be easily implemented or are already in use by some funders but would need wider adoption, deeper reforms that could be conducted by current funding institutions but would replace existing practice, and radical reforms that would require new institutional structures to be developed.

## Incremental Reforms

### Proposal-Centered Funding.

Should the content of the proposals or the reputation of the investigators be accorded more weight? Studies indicate that this is a crucial question. A quasi-experimental study at the Canadian Institutes of Health Research reported that when peer review focused on the scientist (evaluation of leadership, productivity, and vision), 13.9% (199/1,427) of applications by men were funded compared with 9.2% (64/698) of applications by women. In contrast, when the peer review focused on the proposed science, success rates between men and women did not differ significantly (15). Similarly, the NSF conducted experiments and reported that anonymized proposals on evolution and climate change led to strikingly different rankings of the proposals than those of the standard nonanonymous procedure (34).

To the degree that these findings can be generalized, they have been used to argue first that funding agencies should eliminate all questions from their forms that evaluate the principal investigator and instead evaluate the proposal alone, and second that agencies should request anonymized proposals (35). Both actions are likely to reduce unconscious bias with respect to gender and other groups, as well as the bias in path dependence. Counterarguments can also be made against the idea of fully anonymous review. First, it can be almost impossible to maintain anonymity when the field is so small or the researcher so prominent that reviewers can infer which lab is behind the proposal. Second, anonymity is problematic insofar as it may favor researchers who are good at writing proposals but less effective at carrying out the research, and it ignores valuable information about past performance that is predictive of future success or failure. These considerations lean perhaps in favor of a more modest step, of asking reviewers to weight the proposal more strongly but not to completely ignore principal investigator quality. The Australian Research Council (ARC) explicitly assigns 70% weight to the proposal and 30% to the researchers during its review process. The ERC has taken a similar step in this direction by asking, since 2023, its panel members to confer substantially more weight to the proposal than to the principal investigator, thereby modifying its previous policy of equal weights for both. It is too soon, of course, to evaluate the impact of this change.

### Representative Composition of Review Panel Members.

A funding agency can try to counter bias by implementing a systematic selection procedure for panel members that focuses on their representativeness. This can be implemented via a database for potential panel members, which is used to replace retiring members according to gender, race, university, region, or country in order to maintain a representative composition of reviewers in their panels. In the US context, grant review panels for agencies such as NSF are typically convened in an ad hoc manner by the responsible program officers, who may select panelists according to a variety of criteria. Program directors can achieve balance if they have access to an appropriately diverse pool of potential panelists.

The Scientific Council of the ERC selects reviewers on the basis of requests from its executive agency, such as for an “experimental economist, female, not from the United Kingdom, Germany, Spain, and the Netherlands.” Appropriate representation of male and female researchers may have contributed to the ERC achieving a stable equal success rate for men and women. For international funding agencies, representative compositions relative to country can also address the country disparities. In the ERC, however, that measure has thus far not resolved the issue of low participation and success rates of about half of its 27 member states. One reason seems to be that it is difficult to recruit panel members from these countries. Although the more than 100 panels of 16 or more members each are highly international, including reviewers from associated countries such as Israel and Norway and nonassociated countries such as the United States, members from less well-funded countries are hard to find.

The ideal of representative composition also has limits. For example, serving on a review panel places an extra burden on already overworked women in fields in which they are presently underrepresented and can be detrimental to their own research.

### Hosting Researchers from Low-Funding Countries.

A proposal to reduce the country disparities is to invite young researchers from countries with low funding to institutions or labs that are well funded. Invited researchers are thereby connected to research networks and learn firsthand how to conduct fundable research. The ERC implemented two such programs, asking successful grantees whether they would be willing to host young researchers from less successful countries. According to data supplied by the ERCA to the first author of this article, 58 individuals who have participated in the ERC Visiting Fellowship Programmes since 2016 applied for ERC grants. Their success rate was comparable to that of their host institutions. The ERC Individual Mentoring Initiative, which started in 2021, yielded 22 applications from 108 mentees (a total of 94 mentors were involved to support these mentees).

The NSF at present states explicitly that international participants should seek funding from their own countries for collaborative projects with NSF-funded researchers in the United States. A modest recommendation is that the NSF and other funding agencies provide more funding for international collaborations targeting underfunded regions. The US State Department’s Fulbright Scholars program already exists to bring foreign researchers to the United States. Although the State Department and the NSF have different goals, it could be argued that more coordination between these two funding sources could have a favorable impact on the goals of both organizations.

### Systematic Scientific Evaluation of Funding Schemes as a Rule.

Following its mandate from the CHIPS and Science Act of 2022 to consider alternatives to its funding practices, the NSF announced a partnership with the Metascience Working Group of the Institute for Progress (IFP) to design and execute experiments exploring how the agency funds and supports research and innovation. Given the many uncertainties about the effect of current and alternative funding models, one might wish for more experimental research. However, it is difficult to conduct studies that are capable of eliminating all the confounds, and even when experimental studies are conducted, their impact may be ignored.

For instance, in 1995, while serving as members of NIH’s Review of Grant Applications Committee, psychologists Hal Arkes and Robyn Dawes grew concerned about the low reviewer reliability they observed. They found experimentally that when reviewers rated each proposal on several criteria separately as opposed to the existing practice of giving one overall rating number, reviewer consistency was higher (36). Yet when they presented these results to the NIH officials, the reaction “was intensely negative” (36; P. 430).

More recently, most funding agencies, including the NIH, would agree that they should constantly monitor their funding process for potential biases such as success rates by gender, make their data openly accessible, and investigate the causes of potential biases. For instance, in 2019 the ERC created the Committee for Programme Impact Monitoring and Evaluation, which makes quantitative evaluations of completed projects available to the public. More controversial is the potential for experimental investigations to establish the best funding schemes; conducting such studies and convincing administrators to evaluate their practices may be exceedingly difficult.

Some grounds for optimism about both the feasibility of such research and its effect on agencies can be derived from the aforementioned alliance between NSF and IFP. An example is an experimental study by the Patent and Trademark Office (USPTO) evaluating a pilot program that raised successful patent application rates for both men and women and contributed to closing the gender gap by having a larger impact on the success of applications by women (37). This pilot program was subsequently adopted by the USPTO.

## Deeper Reforms

### Distributed Rather than Concentrated Funding.

Should large amounts be distributed to just a few researchers or research centers, or should smaller amounts be distributed across a larger number of researchers and sites? Opting to fund larger grants for fewer projects has been justified on the basis of rewarding and promoting excellent research. Some argue that funding agencies would disincentivize the best researchers if resources were allocated approximately equally to scientists whose productivities differ markedly (38, 39). In fact, the distribution of publications, innovations, patents, and impact across scientists is highly positively skewed. Furthermore, past research performance is a good predictor of future potential (40). National Centers of Excellence are one salient type of concentrated resources, providing focused support for world-class research groups (41), establishing shared and often expensive facilities used by many scientists (42), and creating networks in which scientists naturally learn about and build upon each other’s results (43). Small groups of researchers often fail to achieve the critical mass necessary for interactions among scientists to create transformative organizational “phase transitions” (44). Science often advances by creating major infrastructural resources, such as large telescopes, particle accelerators, supercomputers, or comprehensive databases. These expensive infrastructures would often be impossible to construct and maintain without concentrated funding.

Arguing against such concentration of resources is the finding that, on average, larger budgets and labs have diminishing returns, as measured by publication and citation indicators (45). With the caveats that these indicators are questionable ("*Disallow reporting of metrics*" on metrics, below) and that such studies do not experimentally intervene using randomized controlled trials and thus do not warrant strong causal claims between funding decisions and outcomes for science, most available studies nevertheless observe decreasing marginal gains in scientific impact past a certain point (for a review, see ref. 46). Studies across several nations show that the research output (47), citation-based impact (48), and longevity of impact (49) per dollar of investment decrease as the entire grant amount increases. In some cases, funding amount correlates negatively or not at all with the journal impact per paper (50). While some studies do show positive correlations between scientific impact and amount of funding (51), the common pattern of diminishing returns with increasing grant sizes points to efficacy benefits for small to moderate-sized grants (52, 53). (The large number of scientists engaged in very large projects may, however, actually reflect a lower amount of funding per scientist.) Scientists early in their careers typically have broader and more varied training opportunities when resources are broadly distributed.

More distributed funding means funding more proposals, which in turn could largely take care of the issue of limited reviewer reliability. For instance, if the rankings of the top 2% to 20% proposals are unreliable, and there are diminishing returns past the 20th percentile, (24) then funding 20% or more would reduce this unreliability. The more proposals in the difficult range that can be funded, the less need to discriminate between these. A shift to more distributed funding can also reduce the path dependence effect by supplying grants to more researchers and address some of the issues of country or region disparities. More distributed funding additionally enables agencies to fund a higher diversity of projects, thus leaning toward the “exploration” pole of the explore–exploit continuum, that is, more explorative research searching for new insights rather than exploiting insights that are already known (54). Agencies could conceive separate funding streams with smaller amounts of money and shorter time horizons for proposals that explore ideas. Exploration protects against prematurely converging on local maxima in a solution space (55). The ARC Discovery Project funds exploratory projects with amounts between $30,000 and $500,000, aiming for broad distribution by limiting researchers to no more than two such awards during their career.

A low general cap on the maximum funding per award would guarantee widest possible distribution but comes with disadvantages, such as not providing sufficient funding for expensive projects and disincentivizing top researchers from pursuing excellence. However, a shift to more distributed funding does not require capping all awards at the same level. For instance, ERC advanced grants provide up to 2.5 million euros for 5 y, with a success rate of 13 to 15% (and the PI can request additional funds of up to 1 million euros). One might argue for applicants choosing their own funding amount that fits their project, so that a natural variability arises. Yet virtually all applicants ask for an amount very close to the maximum, meaning that there is no natural diversification. The alternative is a shift to more distributed funding that leaves the top amount as it is but offers the possibility of applying for lower amounts. A minimal change would be to provide at least two types of grants within the same call, one type at the current maximum level and another type at up to half of this amount (or a value chosen near the point of diminishing marginal gains), but with an equal total amount of available funding in both categories. The NIH occasionally offers this possibility by accepting proposals at either the R01 or R21 levels within the same call. This enables more grants to be funded and also provides an incentive to apply for smaller amounts with higher expectations of success. If too many researchers respond to this incentive, however, the net result may be greater competition and lower success rates for the smaller awards.

A moderate position argues for heterogeneity in the amounts offered, with an emphasis toward distributed funding at lower levels for more researchers. In the United States, this might be enhanced by requiring universities, which are both tax-exempt and federally funded through massive grant overheads, to dedicate some portion of these funds to internal seed grants (perhaps beyond what many of them do voluntarily). Finding an effective balance among these approaches invites systematic empirical study that is presently lacking.

### Retrospective Funding: Awards for Completed Science and Prize Competitions.

Given the difficulty in predicting the future success of a proposed research, the opposite approach merits examination: Awarding funds not before but after research has been successfully completed. There are two types of such retrospective funding.

The first awards large grants to a few excellent researchers. This can be in the form of a one-time sum, as with a Nobel Prize, or a permanent research endowment, as with the directors of the Max Planck Institutes (MPIs), or with a multiyear commitment such as the Changjiang Scholar awards in the People’s Republic of China. The philosophy of the MPIs is based on a set of heuristic principles. First, research is built up around leading researchers, not a field. Universities, in contrast, typically select a field and then hire the best person in that field they can attract. The second principle is to take risks and ideally create new fields rather than merely excel in existing fields. To enable such risk-taking, MPI directors annually receive unconditional lump sums to conduct research, independent of the judgment of peer reviewers for proposal-based grant agencies. This allows for long-term planning of research rather than short-term planning from one grant to the next. For more than a century, this funding model has been successful on many measures. Based on quality and trust, the approach circumvents the problems of low reviewer reliability and of having to guess the quality of future outcomes on the basis of a proposal. Yet it can only be one of many funding models because it is limited to a small number of directors. Furthermore, this approach suffers from issues surrounding gender bias, as the overwhelmingly male list of MPI directors suggests (250 out of 304, 82%, in 2021), along with highly publicized complaints about different evaluative standards applied to female MPI directors (56).

The second option consists of prize competitions, also called *research tournaments*. Here, researchers compete with one another to achieve a goal, as in the DARPA Grand Challenges for the design of autonomous vehicles. Prize competitions can remove some of the issues related to prospective funding, such as low reviewer reliability and path dependence. The downsides of prize competitions include that researchers must make up-front investments and accept losses if they do not receive a prize, which reinforces existing biases favoring well-funded labs (57), further intensifying the Matthew effect. Most prize competitions are mission-directed research because the goal needs to be specified precisely by an agency in order to be able to determine the winner (58).

### Partial Lotteries.

A relatively recent development is the allocation of funding through lotteries. Funding-by-lottery is often perceived as a radical alternative to the current merit-based funding schemes due to the explicit introduction of randomness into the funding process. However, in contrast to popular belief, lotteries that completely randomize the selection of proposals are rare. Instead, realistic proposals suggest enriching the peer review process through random selection. Indeed, lotteries are reasonable only if combined with a preceding evaluation process. Such models are called *partial lotteries*. Although the NSF, the UK Research and Innovation (UKRI), the French National Research Agency (ANR), the DFG, and the ERC have explored partial randomization, none of them has implemented it thus far. There are valid concerns that downweighing the importance of peer review in the selection process may negatively affect public trust in the research funding system and frustrate researchers’ desire for merit-based funding (59, 60).

There are two kinds of partial lotteries, two-step and three-step procedures. In a two-step partial lottery, reviewers first weed out weak projects but do not rank the strong ones (61). In the second step, the remaining subset of strong projects are funded at random until the budget is exhausted. In 2013, the Health Research Council of New Zealand was the first major funding agency to implement such a two-step procedure for their Explorer Grant scheme, which seeks to fund transformative research at an early stage (62). In Step 1, short and anonymized applications (six pages) are rated by panel members on whether they are potentially transformative and also viable, that is, have the potential to create a new paradigm or pathway. In Step 2, applications that are rated both viable and potentially transformative are assigned random numbers and are selected in order until the budget is exhausted. A survey of 126 applicants found that 63% considered partial lotteries acceptable for this kind of exploratory grant, but only 40% favored them for other types of grants (62). Acceptance was, of course, highest among those fortunate researchers who had won a grant via partial lottery.

The relative economic value of conventional contest-based funding versus a two-step partial lottery was assessed in a simulation study (61). The simulations indicated that contest-based funding becomes inefficient when the number of possible awards exceeds the number of strong proposals. In this case, researchers invest disproportionately more effort in proposal writing than in actual research. Lotteries can restore efficiency as funding lines decline, albeit at the cost of reducing the average scientific value of funded projects (61).

The alternative is a three-step partial lottery based on the previously mentioned empirical observation that reviewers could identify the top 2% of proposals (24). In a three-step scheme, Step 1 is the same as in the two-step scheme, where reviewers filter out all weak proposals. Step 2 is different: Among the strong proposals, reviewers pick the very best projects for which there is a consensus, which may be 1 to 5%, and fund these. Finally, in Step 3, a lottery is applied to the remaining strong proposals. The Volkswagen Foundation (63) has piloted such a three-step program.

The argument in favor of partial lotteries is that they reduce several of the issues of current funding regimes, including gender bias and other forms of discrimination, as well as path dependence. They also explicitly address the issue of the discriminability of the top proposals, with the exception of the very top ones. Rather than relying on extrinsic factors such as the gender, race, name, or institution of the applicant, a partial lottery explicitly admits the partial role of chance in the process. Finally, partial lotteries can strongly reduce the time panel members spend on discussing the ranking of strong proposals. At the same time, pilot studies indicate that applicants spend an equal amount of time on writing proposals, whether or not these include partial lotteries (61). Three-step partial lotteries are arguably superior to the two-step version because they ensure that proposals identified by all reviewers as the very best are actually funded. However, because these schemes involve reviewer judgments, the Matthew effect and other biases associated with such judgments are not entirely eliminated.

### Disallow Reporting of Metrics.

A panel in a large funding agency may have to deal with hundreds of applications for each call and spend some 4 to 5 full days discussing these, not to mention the time spent on written reviews. And there may be dozens of such panels for each call. Given such a workload, some panel members may be tempted to rely heavily or exclusively on metrics rather than on the content of the proposal. Metrics, however, evaluate solely the principal investigator, not the proposal. In the extreme, when only h-index and the number of publications times impact factors are calculated, the content of the proposed research becomes irrelevant. As already discussed, the focus on the applicant promotes gender bias and possibly also path dependence of funding.

There are two types of metrics, individual metrics (such as the h-index of an individual researcher) and surrogate metrics (such as the impact factor of a journal in which a researcher published). The most radical alternative is that funding agencies require applicants to exclude any metrics such as citation rates, h-index, impact factors, and number of publications in their proposals. Excluding this will of course not prevent reviewers from looking up the information on the internet, but it sends a signal to both reviewers and applicants that the content of the proposal should be evaluated, not metrics. Because most agencies want to base funding decisions on both the applicant and the proposal, this alternative is viable only if the funding agency additionally asks the applicants to submit a small number of what they consider their most important publications or patents. Reviewers can then gain a picture of the content of the applicant’s previous work, rather than looking at numbers only. We are not aware of any large funding agency that has implemented this radical alternative of disallowing all metrics.

A more moderate alternative is to explicitly prohibit applicants from reporting surrogate metrics such as journal impact factors, that is, metrics that directly concern not the performance of the applicant but that of a journal (and which can be inflated by various measures). This is promoted by the San Francisco Declaration on Research Metrics (64), thereby illustrating the degree to which overreliance on metrics is well known in some quarters.

Metrics such as impact factors were developed by librarians for their purposes, not for measuring the quality of individual researchers. Yet such metrics have become an administrative surrogate for judgment of quality. A shift away from impact factors and other metrics appeals to many scientists who perceive a need to liberate science from the hands of journal publishers and administrators.

Goodhart’s law states that any measure that becomes a target ceases to be a good measure. In the context of scientific research, this law may also be expressed as a concern that rewarding what conforms to the existing metrics favors what is already known and thereby disincentivizes truly innovative research.

### The Use of Generative AI in Proposal Evaluation.

Some researchers have suggested that reviewing could be made a less onerous task through the use of large language models (LLMs) such as ChatGPT, and many scientists are already using LLMs to speed up writing proposals (65). Here, we note briefly that the NIH, the NSF, and the ARC have explicitly adopted policies forbidding the use of LLMs for the purpose of reviewing (66–68). Uploading grant proposals to LLMs is already illegal in the EU under existing laws. The companion piece in this issue by Binz et al. provides a detailed discussion of the use of generative AI in proposal evaluation.

## Radical Alternatives

### Removal of Political Governance.

Former president of the ERC Helga Nowotny noted in an interview with *Nature* in November 2010 that the problems at the ERC “all boil down to one main problem—that of governance. We do not have the independence to run the ERC the way a frontier research agency should ideally be run” (69). The European Commission, which established the ERC, tends to squeeze it into the same administrative straitjacket as the other parts of the Commission. The independence of national agencies is also contingent on national politics. The issue of control is not about which proposals are funded but about the agency’s freedom to organize the process of funding in an efficient way. This freedom entails, for instance, freedom from an administration’s preference for complex forms for applications, time-consuming reporting duties for spending funds (see The trade-off between writing proposals and managing grants versus doing research), and micromanaging details of resource management. Governance is also a main issue for enabling systematic evaluation of alternative models of funding, and for actual funding reform. Furthermore, politicians tend to shift funding away from curiosity-driven science and research with long-term or unforeseeable payoffs, and this can shape the intellectual landscape by favoring research that is deemed to be in the national interest and steering young researchers toward these topics. Either tendency could harm the progress of science.

The first radical alternative to the status quo is to liberate all large funding agencies from political and administrative influence, and let science be governed by scientists. Proponents of this approach can point to Max Planck Society’s philosophy of trusting their directors, and they may argue that freedom is not an untenable risk but a precondition for successful science. Opponents of this approach can point to historical examples of failed self-governance, where unethical research was pursued by scientists working without adequate oversight. A further argument against complete freedom from political control stems from the idea that voters in a democratic society have a right to say how public money should be spent, even while it is important to protect research from politicization (70).

Reducing political governance of public funding agencies does not mean removing governmental funding and oversight altogether. However, an even more radical proposal comes from those who claim that scientific and other intellectual freedom exists only when government has no influence at all, and that research funding should therefore be left entirely to the free market. Underlying this view is also the claim that the free market would be more efficient at allocating funding to research. A counterargument comes even from within industry. Nathan Myhrvold, the creator of Microsoft Research, argued that it would be naïve to believe profit-driven companies would fill the void and altruistically fund curiosity-driven science; instead, most of it would grind to a halt (71) and would be dependent on private donors. In one sense this would be a return to how things were before the institutionalization of public funding, although the existence of private charitable foundations in both Europe and the United States that support curiosity-driven research was absent prior to the 20th century. There is a complicated interaction across different timescales between public and private funding that demands further study of how to balance provision of adequate resources and freedom to innovate (72, 73).

### World Research Council.

Most public funding of curiosity-driven research is at a national level. International funding agencies, in contrast, fund applications from researchers from a number of countries. The ERC is such an international agency, where researchers working in all current 27 EU member states can competitively apply for funding. Its prestige has led non-EU countries to join, including Israel, Norway, and Switzerland, or rejoin, as the United Kingdom. Countries even further afield, such as Canada and Australia, have indicated their interest in joining the ERC. Since its inception in 2007, the ERC has funded 13,000 principal investigators and 90,000 researchers in over 900 institutions, and their grantees have subsequently won 14 Nobel prizes.

This success suggests thinking even bigger and considering a World Research Council (WRC). It could be modeled after the ERC, but with optional membership open to all countries in the world. One might ask, who would pay for a WRC? Like the ERC, funding would be provided by the member states, and it would coexist with national funding agencies, thus allowing countries to maintain their own funding priorities. Science at the highest level has always been international, and the fundamental goal of the WRC would be to establish worldwide collaboration, competition between the best ideas, and the highest standards for research. Free from national and regional preferences, a WRC could implement merit-based funding criteria globally, promoting fair and unbiased distribution of funding around the world. It could also be especially important for addressing geographical funding disparities.

The key to making such an ambitious plan work is governance. The danger is that the issues the ERC faces regarding its independence from the administration of the European Commission could be multiplied. It can be argued that a WRC requires a legal framework that guarantees its governance by scientists alone so that it can work independently of political interests and administrative control and micromanaging. That level of independence requires politicians to trust scientists, which would be difficult and probably result in constant friction, not to mention the obligations of scientists to provide for democratic oversight of public spending and the challenges of working with very diverse ethical standards and requirements. Nevertheless, a WRC could help establish excellence as the only criterion for funding curiosity-driven research worldwide.

A first step toward a worldwide funding agency might be an OECD-wide Research Council (ORC). That would have the advantage of integrating a set of economically more homogeneous countries to help increase their capacity for curiosity-driven research. At the same time, it would still include countries that so far have shown little activity in applying for funding, such as Hungary, Portugal, and Mexico, as well as countries such as India and Indonesia that are mostly excluded from the agencies discussed above. Note that an ORC would not mean terminating all national funding schemes. Rather, it would complement national agencies, which could lead to mutual learning and cumulative knowledge. International funding agencies, be they worldwide or OECD-wide, could better resolve the issue of disparity between countries than do national agencies. They could implement a balanced composition by country and gender of reviewer panels and of its Scientific Council at the governance level. And there is a larger vision: If there is any hope for worldwide peaceful cooperation, it is provided by the scientific community (74).

## Conclusion

The goal of this paper has been to survey issues and arguments for and against various reforms to current procedures for funding curiosity-driven science. We hope to have provided a resource for future deliberation about science funding. Some of the more radical suggestions for reform may be controversial, but it has not been our goal to provide a consensus statement about the issues concerning science funding or about any of the approaches. Regardless of how valid the individual authors and readers deem these various issues and proposals, the success of funding mechanisms for curiosity-driven research should never be taken for granted. Reimagination of funding is essential to the progress of science.

## Data Availability

Data on the ERC Visiting Fellowship Programmes (since 2016) and data on the ERC Individual Mentoring Initiative (since 2021) can be requested from the ERCEA (Xenia.RAJEWSKY@ec.europa.eu).

## References

[1] European Commission, Mission-directed research and innovation: Inventory and characterization of initiatives (2024). https://op.europa.eu/en/publication-detail/-/publication/3b46ce3f-5338-11e8-be1d-01aa75ed71a1/language-en. Accessed 19 November 2024.

[2] J. P. Myklebust, Should the ERC be Worried about its Low Success Rate? (University World News, 2020).

[3] European Research Council, Assessing the influence of ERC-funded research on patented inventions (2022). https://erc.europa.eu/sites/default/files/2023-01/Assessing_the_Influence_ERC-funded_Research_Patented_Inventions.pdf. Accessed 19 November 2024.

[4] R. S. Harrison, T. Barnard Ross, L. Pregelj, Restoring Australia’s long-term innovation requires investment in basic research. Microbiol. Aust. **44**, 57–61 (2023).

[5] J. Reb, S. Luan, G. Gigerenzer, Smart Management: How Simple Heuristics Help Leaders Make Good Decisions in an Uncertain World (MIT Press, 2024).

[6] M. Bahar, R. Griesbach, Can a technology transfer office make a difference in increasing licensing numbers: Incorrect assumptions and inadequate context? LES Nouv. **LIII**, 132–238 (2018).

[7] A. Mossoff, A. Adalja, Patents as a driver of the unprecedented biomedical response to COVID-19. Inquiry **59**, 00469580221124819 (2022). 36129280 10.1177/00469580221124819 PMC9500249

[8] B. Bozeman, D. Sarewitz, Public value mapping and science policy evaluation. Minerva **49**, 1–23 (2011).

[9] Z. Pirtle, J. Moore, Where does innovation come from? IEEE Technol. Soc. Mag. **38**, 56–67 (2019).

[10] M. Szell, R. Sinatra, Research funding goes to rich clubs. Proc. Natl. Acad. Sci. U.S.A. **112**, 14749–14750 (2015). 26567155 10.1073/pnas.1520118112 PMC4672774

[11] T. Bol, M. de Vaan, A. van de Rijt, The Matthew effect in science funding. Proc. Natl. Acad. Sci. U.S.A. **115**, 4887–4890 (2018). 29686094 10.1073/pnas.1719557115 PMC5948972

[12] UK Research & Innovation, Regional distribution of UKRI spend. *UK Research and Innovation* (2021). https://www.ukri.org/wp-content/uploads/2021/03/UKRI-240321-RegionalFunding20182019-AnalysisReport.pdf. Accessed 19 November 2024.

[13] M. Hourihan, Towards a solution for broadening the geography of NSF funding. Federation of American Scientists (2024). https://fas.org/publication/towards-a-solution-for-broadening-the-geography-of-nsf-funding/. Accessed 19 November 2024.

[14] L. Husu, S. de Cheveigné, "Gender and gatekeeping of excellence in research funding: European perspectives" in Gender Change in Academia, Re-Mapping the Fields of Work, Knowledge, and Politics from a Gender Perspective, B. Riegraf, B. Aulenbacher, E. Kirsch-Auwärter, U. Müller, Eds. (Springer, 2010), pp. 43–59.

[15] H. O. Witteman, M. Hendricks, S. Straus, C. Tannenbaum, Are gender gaps due to evaluations of the applicant or the science? A natural experiment at a national funding agency. Lancet **393**, 531–540 (2019). 30739688 10.1016/S0140-6736(18)32611-4

[16] R. Van der Lee, N. Ellemers, Gender contributes to personal research funding success in The Netherlands. Proc. Natl. Acad. Sci. U.S.A. **112**, 12349–12353 (2015). 26392544 10.1073/pnas.1510159112 PMC4603485

[17] L. Bornmann, R. Mutz, H.-D. Daniel, Gender differences in grant peer review: A meta-analysis. J. Inform. **1**, 226–238 (2007).

[18] S. E. Waisbren , Gender differences in research grant applications and funding outcomes for medical school faculty. J. Womens Health **17**, 207–214 (2008). 10.1089/jwh.2007.0412 18321172

[19] K. Hermansson, C. Jacobsson, R. Österberg, Gender equality in research funding. A study of 11 European countries, Israel, and Canada (2021). https://gender-net-plus.eu/wp-content/uploads/2021/04/GNP-Deliverable-D6.3-Gender-Equality-in-Research-Funding-plus-Country-reports-final.pdf. Accessed 19 November 2024.

[20] M. Ranga, N. Gupta, H. Etzkowitz, Gender effects in research funding. Deutsche Forschungsgemeinschaft (2012). https://www.dfg.de/resource/blob/170570/e48fab44b49274b83e2b5aeb382145d0/studie-gender-effects-data.pdf. Accessed 19 November 2024.

[21] R. Beck, V. Halloin, Gender and research funding success: Case of the Belgian F.R.S.-FNRS. Res. Eval. **26**, 115–123 (2017).

[22] E. A. Erosheva , NIH peer review: Criterion scores completely account for racial disparities in overall impact scores. Sci. Adv. **6**, eaaz4868 (2020). 32537494 10.1126/sciadv.aaz4868 PMC7269672

[23] A. C. Morgan , The unequal impact of parenthood in academia. Sci. Adv. **7**, eabd1996 (2021). 33627417 10.1126/sciadv.abd1996 PMC7904257

[24] D. Li, L. Agha, Big names of big ideas: Do peer-review panels select the best science proposals? Science **348**, 434–438 (2015). 25908820 10.1126/science.aaa0185

[25] F. C. Fang, A. Bowen, A. Casadevall, Research: NIH peer review percentile scores are poorly predictive of grant productivity, eLife **16**, 13323 (2016). 10.7554/eLife.13323 PMC4769156 26880623

[26] E. L. Pier, M. Brauer, A. Filut, M. Carnes, Low agreement among reviewers evaluating the same NIH grant applications. Proc. Natl. Acad. Sci. U.S.A. **115**, 2952–2957 (2018). 29507248 10.1073/pnas.1714379115 PMC5866547

[27] D. Cicchetti, The reliability of peer-review for manuscript and grant submissions—It’s like deja-vu all over again—Author’s response. Behav. Brain Sci. **16**, 401–403 (1993).

[28] M. Fogelholm , Panel discussion does not improve reliability of peer review for medical research grant proposals. J. Clin. Epidemiol. **65**, 47–52 (2012). 21831594 10.1016/j.jclinepi.2011.05.001

[29] S. Guthrie , Measuring bias, burden and conservatism in research funding processes. F1000 Research **8**, 851 (2019).

[30] E. L. Pier , ‘Your comments are meaner than your score’: score calibration talk influences intra- and inter-panel variability during scientific grant peer review. Res. Eval. **26**, 1–14 (2017). 28458466 10.1093/reseval/rvw025 PMC5407376

[31] F. C. Fang, A. Casedevall, Research funding: The case for a modified lottery. mBio **7**, e00422-16 (2016). 27073093 10.1128/mBio.00422-16 PMC4959526

[32] B. Aczel , The present and future of peer review: Ideas, interventions, and evidence. Proc. Natl. Acad. Sci. U.S.A. (this issue). 10.1073/pnas.2401232121 PMC11804526 39869808

[33] P. Crushman , "Impact of declining proposal success rates on scientific productivity" in AAAC Meeting (2015). http://arxiv.org/pdf/1510.01647. Accessed 19 November 2024.

[34] Y. Bhattacharjee, NSF’s ‘big pitch’ tests anonymized grant reviews. Science **336**, 969–970 (2012). 22628627 10.1126/science.336.6084.969

[35] M. Lauer, Anonymizing peer review for the NIH directors’ transformative research award application. https://nexus.od.nih.gov/all/2020/05/27/anonymizing-peer-review-for-the-nih-directors-transformative-research-award-applications/. Accessed 19 November 2024.

[36] H. R. Arkes, V. A. Shaffer, R. M. Dawes, Comparing holistic and disaggregated ratings in the evaluation of scientific presentations. J. Behav. Decis. Mak. **19**, 429–439 (2006).

[37] N. Pairolero, A. A. Toole, P.-A. Pappas, C. deGrazia, M. Teodorescu, Closing the gender gap in patenting: Evidence from a randomized control trial at the USPTO. Academy of Management Proceedings (2022). https://journals.aom.org/doi/epdf/10.5465/AMBPP.2022.197. Accessed 19 November 2024.

[38] D. Hicks, J. S. Katz, Equity and excellence in research funding. Minerva **49**, 137–151 (2011).

[39] K. Vaesen, J. Katzav, How much would each researcher receive if competitive government research funding were distributed equally among researchers? PLoS ONE **12**, e0183967 (2017). 28886054 10.1371/journal.pone.0183967 PMC5590858

[40] B. Gyórffy, P. Hermann, I. Szabó, Research funding: Past performance is a stronger predictor of future scientific performance than reviewer scores. J. Informatics **14**, 101050 (2020).

[41] T. Ida, N. Fukuzawa, Effects of large-scale research funding programs: A Japanese case study. Scientometrics **94**, 1253–1273 (2013).

[42] A. Bonaccorsi, C. Daraio, Exploring size and agglomeration effects on public research productivity. Scientometrics **63**, 87–120 (2005).

[43] T. Hellström, L. Jabrane, E. Brattström, Center of excellence funding: Connecting organizational capacities and epistemic effects. Res. Eval. **27**, 73–81 (2017).

[44] R. Kenna, B. Berche, Critical mass and the dependency of research quality on group size. Scientometrics **86**, 527–540 (2017).

[45] J. Basson, L. Lorsch, T. Dorsey, Revisiting the dependence of scientific productivity and impact on funding level. *NIH General Medicine Sciences* (2016). https://loop.nigms.nih.gov/2016/07/revisiting-the-dependence-of-scientific-productivity-and-impact-on-funding-level/. Accessed 19 November 2024.

[46] K. Aagaard, A. Kladakis, M. W. Nielsen, Concentration or dispersal of research funding? Quant. Sci. Stud. **1**, 117–149 (2020).

[47] A. Asonuma, H. Urata, "Academic funding and allocation of research money" in The Changing Academic Profession in Japan, A. Arimoto , Eds. (Springer, 2015), pp. 57–77.

[48] M. Doyle , Association of percentile ranking with citation impact and productivity in a large cohort of de novo NIMH-funded R01 grants. Mol. Psychiatry **20**, 1030–1036 (2015). 26033238 10.1038/mp.2015.71 PMC5526589

[49] C. Bloch, A. Kladakis, M. P. Sørensen, “Size matters! On the implications of increasing the size of research grants” in Handbook of Public Research Funding, B. Lepori, D. Jongbloed, D. Hicks, Eds. (Edward Elgar Publishing, 2023), pp. 123–138.

[50] H. Jung, I. Seo, J. Kim, B.-K. Kim, Factors affecting government-funded research quality. Asian J. Technol. Innovation **25**, 447–469 (2017).

[51] E. Yan, C. Wu, M. Song, The funding factor: A cross-disciplinary examination of the association between research funding and citation impact. Scientometrics **115**, 369–384 (2018).

[52] M. S. Lauer, N. S. Danthi, J. Kaltman, C. Wu, Predicting productivity returns on investment. Circ. Res. **117**, 239–243 (2015). 26089369 10.1161/CIRCRESAHA.115.306830 PMC4506707

[53] P. Mongeon, C. Brodeur, C. Beaudry, V. Larivière, Concentration of research funding leads to decreasing marginal returns. Res. Eval. **25**, rvw007 (2016).

[54] T. T. Hills , Exploration versus exploitation in space, mind, and society. Trends Cogn Sci. **19**, 46–54 (2015). 25487706 10.1016/j.tics.2014.10.004 PMC4410143

[55] M. Galesic , Beyond collective intelligence: Collective adaptation. J. R. Soc. Interface **20**, 20220736 (2023). 36946092 10.1098/rsif.2022.0736 PMC10031425

[56] A. Abbot, Scientists question Max Planck Society’s treatment of women leaders. Nature **600**, 20 (2021).

[57] P. Stephan, How Economics Shapes Science (Harvard University Press, 2015).

[58] Office of Science Technology and Policy Implementation of Federal Prize and Citizen Science Authority: Years 2019–20 (2022). https://www.whitehouse.gov/wp-content/uploads/2022/05/05-2022-Implementation-of-Federal-Prize-and-Citizen-Science-Authority.pdf. Accessed 19 November 2024.

[59] J. Shaw, Peer review in funding-by-lottery: A systematic overview and expansion. Res. Eval. **32**, 86–100 (2023).

[60] M. Reinhart, C. Schendzielorz, The lottery in Babylon—On the role of chance in scientific success. J. Responsible Innov. **7**, S25–S29 (2020).

[61] K. Gross, C. T. Bergstrom, Contest models highlight inherent inefficiencies of scientific funding competitions. PLoS Biol **17**, e3000065 (2019). 30601806 10.1371/journal.pbio.3000065 PMC6314589

[62] M. Liu , The acceptability of using a lottery to allocate research funding: A survey of applicants. Res. Integr. Peer Rev. **5**, 3 (2020). 32025338 10.1186/s41073-019-0089-z PMC6996170

[63] Volkwagen Foundation, Experiment! – In search of bold research ideas (2017). https://www.volkswagenstiftung.de/en/funding/funding-offer/experiment-search-bold-research-ideas-completed. Accessed 19 November 2024.

[64] DORA, The San Francisco Declaration on Research Assessment. *DORA* (2012). https://sfdora.org/read//read/. Accessed 19 November 2024.

[65] R. Van Noorden, J. M. Perkel, AI and science: What 1,600 researchers think. Nature **621**, 672–675 (2023). 37758894 10.1038/d41586-023-02980-0

[66] NIH, The use of generative artificial intelligence technologies is prohibited for the NIH peer review process (2023). https://grants.nih.gov/grants/guide/notice-files/NOT-OD-23-149.html. Accessed 19 November 2024.

[67] NSF, Notice to research community: Use of generative artificial intelligence technology in the NSF merit review process (2022). https://new.nsf.gov/news/notice-to-the-research-community-on-ai. Accessed 19 November 2024.

[68] J. Kaiser, Science funding agencies say no to using AI for peer review. Science **381**, 261 (2023). 37471562 10.1126/science.adj8309

[69] A. Abbott, European Research Council battles bureaucracy. Nature (2010). https://www.nature.com/articles/news.2010.615. Accessed 19 November 2024.

[70] H. Douglas, The Rightful Place of Science: Science, Values, and Democracy. The 2016 Descartes Lectures (Consortium for Science, Policy & Outcomes, 2021).

[71] N. Myhrvold, Basic science can’t survive without government funding. Sci. Am. **314**, 11 (2016).

[72] B. Becker, Public R&D policies and private R&D investment: a survey of the empirical evidence. J. Econ. Surv. **29**, 917–942 (2015).

[73] N. Bloom, J. Van Reenen, H. Williams, A toolkit of policies to promote innovation. J. Econ. Perspect. **33**, 163–184 (2019).33343082

[74] L. Daston, Rivals., How Scientists Learned to Cooperate (Columbia Global Reports, 2023).

